# CNEFinder: finding conserved non-coding elements in genomes

**DOI:** 10.1093/bioinformatics/bty601

**Published:** 2018-09-08

**Authors:** Lorraine A K Ayad, Solon P Pissis, Dimitris Polychronopoulos

**Affiliations:** 1 Department of Informatics, King’s College London, London, UK; 2 Genomics England, Charterhouse Square, London, UK

## Abstract

**Motivation:**

Conserved non-coding elements (CNEs) represent an enigmatic class of genomic elements which, despite being extremely conserved across evolution, do not encode for proteins. Their functions are still largely unknown. Thus, there exists a need to systematically investigate their roles in genomes. Towards this direction, identifying sets of CNEs in a wide range of organisms is an important first step. Currently, there are no tools published in the literature for systematically identifying CNEs in genomes.

**Results:**

We fill this gap by presenting CNEFinder; a tool for identifying CNEs between two given DNA sequences with user-defined criteria. The results presented here show the tool’s ability of identifying CNEs accurately and efficiently. CNEFinder is based on a *k*-mer technique for computing maximal exact matches. The tool thus does not require or compute whole-genome alignments or indexes, such as the suffix array or the Burrows Wheeler Transform (BWT), which makes it flexible to use on a wide scale.

**Availability and implementation:**

Free software under the terms of the GNU GPL (https://github.com/lorrainea/CNEFinder).

## 1 Introduction

Conserved non-coding elements (CNEs) are a pervasive class of elements that are usually identified by inspecting whole-genome alignments between two or more genomes. CNEs can be extremely conserved across evolution, yet they do not encode for proteins. Some of these elements play roles in the development of multicellular organisms acting as enhancers (Aparicio *et al.*, 1995). Although they can be referred to in the literature with different names (UCEs, UCNEs, CNS, to name a few), the prevailing view is that these sets of elements are largely overlapping, with their genesis, functions and evolutionary dynamics being largely unknown. We refer the interested reader to (Polychronopoulos *et al.*, 2014a, 2017) for a concise introduction on CNEs.

CNE identification methods may be classified into two major categories: alignment-based and alignment-free methods.

### 1.1 Alignment-based methods

Alignment-based methods identify CNEs by inspecting pairwise or multiple whole-genome alignments. Several tools exist that generate whole-genome alignments, such as BLASTZ (Schwartz *et al.*, 2003), MULTIZ (Blanchette *et al.*, 2004), and LASTZ (Harris, 2007). For a pair of sequences, CNEs are defined as elements which satisfy specific length and sequence identity percentage thresholds (Bejerano *et al.*, 2004; Dubchak *et al.*, 2000; Sandelin *et al.*, 2004b). The threshold values depend on the evolutionary distance between species under comparison. Not all CNEs identified by whole-genome comparisons of mammalian genomes appear conserved when the same conservation criteria are used on more distant genome comparisons. Thus, those thresholds are somewhat arbitrary.

### 1.2 Alignment-free methods

Alignment-free methods avoid some of the problems associated with whole-genome alignments, such as computational complexity, highly fragmented assemblies, and inflexibility. Variants of BLAST are usually used in the homology search on repeat- and coding sequence-masked genomes (Babarinde and Saitou, 2016). Warnefors *et al.* (2016) proposed an alignment-free method based on *k*-mers. All *k*-mers occurring a single time in the reference genome are mapped to the species of interest with a short-read aligner and then overlapping hits are merged into longer CNEs. This approach increases the sensitivity of CNE detection by overcoming the ambiguities and errors in the alignment, such as gap insertions and occurrences of a split across alignment blocks. However, this approach incurs a small false positive rate due to mishandling of hits with multiple copies, either from genome duplications or assembly errors. Most importantly, and similar to many other cases, the authors do not make their implementation for identifying CNEs publicly available.

### 1.3 CNE databases

There also exist many CNE databases which contain already pre-computed sets of CNEs: Ancora (Engström *et al.*, 2008), CEGA (Dousse *et al.*, 2015), cneViewer (Persampieri *et al.*, 2008), CONDOR (Woolfe *et al.*, 2007), UCbase (Lomonaco *et al.*, 2014), UCNEbase (Dimitrieva and Bucher, 2013), and VISTA (Visel *et al.*, 2007). On the one hand, this highlights the importance of this research topic among the biological community. On the other hand, these databases are static and seldom updated. Furthermore, the sets of CNEs stored in these databases are identified using custom scripts, written in different programming languages, and tailored to the biological needs of each study.

### 1.4 Our contribution

In summary, we would like to stress the need for comprehensive tools for identifying CNEs. We present CNEFinder, a tool for identifying CNEs between two given DNA sequences with user-defined criteria. CNEFinder applies the *k*-mer technique of Khiste and Ilie (2015) for computing maximal exact matches. Hence it *does not* require or compute the whole-genome alignment of the two sequences; it *does not* require or compute a whole-genome index such as the suffix array or the BWT—see (Kurtz *et al.*, 2004), for instance—and it thus finds CNEs from the two sequences directly with user-defined criteria. We have designed CNEFinder in a way that we hope proves useful for the biological community: the tool identifies all CNEs around genes of interest with the aim to facilitate functional experiments. Genome- or chromosome-wide CNE trends may also be revealed as demonstrated by our results. We anticipate that CNEFinder will be a useful tool towards cracking the still largely enigmatic regulatory code of our genome.

## 2 Materials and methods

CNEFinder was implemented in the C++ programming language with OpenMP API for multi-platform shared-memory parallel programming. Our implementation (along with a several-page documentation) is distributed under the GNU General Public License (GPL), and is made freely available at https://github.com/lorrainea/CNEFinder.

Given two DNA sequences, a reference sequence *x* and a query sequence *y*, CNEFinder uses the state-of-the-art *k*-mer method presented by Khiste and Ilie (2015), in conjunction with the well-known *seed and extend* strategy (Altschul *et al.*, 1990; Kurtz *et al.*, 2004; Pearson, 2000), to identify CNEs between *x* and *y*. The DNA sequences are first pre-processed to remove exons and simple and low-complexity repeats from the search, allowing the tool to search for CNEs more accurately. CNEs can then be identified by searching the intergenic and intronic regions around a specific gene as input by the user. CNEs can also be identified through the input of specific index positions of chromosomes that exist in the pair of DNA sequences. Moreover, CNEFinder is able to search for CNEs in entire chromosomes. CNEFinder uses a three-stage approach, specifically tailored for CNE identification, described in detail below.

### 2.1 Identifying matches

The *k*-mer-based method (Khiste and Ilie, 2015) for identifying *maximal exact matches* between two sequences is used to identify exact matches (or anchors) between *x* and *y*. The *k*-mers of *x* are first computed using standard bitwise operations; they are then hashed using double hashing; and, finally, they are stored. The corresponding *k*-mers in *y* are then matched using the stored hash table. Attempts to extend the matches of all occurrences of stored positions in the table are carried out. These positions are then returned as maximal exact matches.

We measure the identity score between two strings using the simple edit distance model (Levenshtein, 1966). In this model, the total number of unit-cost edit operations required to transform one string into the other is minimised. The considered operations are insertions, deletions or substitutions of letters. Given a lower bound ℓ on the length of the reported elements and a lower bound t∈(0,1] on the relative identity threshold between two elements (1 returns identical substrings in *x* and *y*), maximal exact matches of minimum length ⌊ℓ/(ℓ−t×ℓ+1)⌋ are computed via applying the *k*-mer-based method (Khiste and Ilie, 2015). This ensures for *exact* matches to be identified, which can then be extended, such that each pair of elements with minimum length ℓ can have an edit distance of at most ℓ−t×ℓ. This follows from a simple counting argument. The user can alternatively set an explicit value for this minimum length, and then the maximum of the two values is considered for the computation.

### 2.2 Merging matches

The anchors found are then merged to produce co-linear sequences of non-overlapping matches and processed further if the combined length of the matches is above a lower bound of nucleotides set by the user with respect to ℓ. The exact identity score at each merging step is calculated by considering the total edit distance of the gaps between the anchors to be merged. The merging process is terminated once the addition of another gap would force the relative identity score to drop below threshold *t*. For edit-distance computation, we apply the fast bit-vector algorithm by Myers (1999). Note that this algorithm applies only for simple edit distance.

### 2.3 Extending matches

The last stage is to check whether the merged matches can be further extended to the left or to the right. At each step of the extension process, the edit distance of the extension in the left and right direction of the merged matches is computed using Myers’ algorithm (Myers, 1999). The current match is extended in both directions if the threshold allows it or otherwise in the direction having the smallest edit distance. This procedure is repeated until the computed relative identity score of the current length of the match reaches *t* or when the maximum length *u* of one of the elements, which is defined by the user, has been reached.

Note that due to the way the extension stage works, the estimated identity score may not be the actual identity score for the whole element: the estimated score could be smaller or equal to the actual. To re-adjust and allow for further extension, the actual identity score is computed for the whole element using Myers’ algorithm (Myers, 1999), and the extension process continues accordingly. Those matches that are of length at least ℓ and at most *u* with relative identity score of at least *t* are reported as CNEs.

## 3 Results

All datasets and output files referred to in this section can be found at https://github.com/lorrainea/CNEFinder. To demonstrate the *accuracy* and *efficiency* of CNEFinder we have conducted the following experiments on a standard desktop PC with an Intel Core i7-4790 CPU at 3.60 GHz with 16 GB of RAM running a GNU/Linux operating system. All optional parameters were set as default unless stated otherwise.

### 3.1 CNEFinder against UCNEbase

The first experiment carried out was to identify how accurate CNEFinder is in computing CNEs by comparing against previously identified CNEs stored in the UCNEbase (Dimitrieva and Bucher, 2013), a well-established CNE database. Specifically, this experiment involved identifying CNEs within five different genes between the Human (hg19) and Chicken (galGal3) genomes. Six different length ranges in base pairs (bp) were tested to identify whether CNEFinder obtained the same CNEs as those present in the UCNEbase. A relative identity threshold of t=95% was used for all datasets. The results show that CNEFinder identifies almost all elements listed in UCNEbase for these datasets and parameters. Table 1 shows the number of CNEs output by CNEFinder that are overlapping with those stored in the UCNEbase. The table presents these results in the form α/β, where α represents the number of CNEs computed by CNEFinder that were found in the UCNEbase, and β the number of CNEs with a length within the specified range stored in the UCNEbase. We only compute the overlap of the identified CNEs against those in UCNEbase as a precision test as there is no ideal set of CNEs to compare against. This overlap analysis is shown in Table 1 using the average percentage of overlapping nucleotides between the CNEs output by CNEFinder and those stored in the UCNEbase. It was computed using the BEDTools Suite (Quinlan and Hall, 2010). Note that the majority of CNEs found by CNEFinder were in fact *longer* in length (bp) than those in the UCNEbase, in addition to having a high overlap percentage for all genes at all length ranges. The full list of identified CNEs can be found online in the same location as the datasets.

**Table 1.** CNEs identified for five genes for different length ranges and t=95%

	200−250 bp		250−300 bp		300−350 bp	
			
	# CNEs	% Nucleotides	# CNEs	% Nucleotides	# CNEs	% Nucleotides
Gene	Overlapping	Overlapping	Overlapping	Overlapping	Overlapping	Overlapping
ZEB2	31/31	84.59	18/18	87.31	20/20	90.36
TSHZ3	35/36	78.49	16/17	80.01	8/8	84.85
EBF3	28/28	87.90	17/17	90.81	16/16	88.21
BCL11A	20/20	81.24	28/28	85.75	14/14	93.61
ZFHX4	18/18	88.02	22/22	89.82	10/10	86.86

	350−400 bp		400−450 bp		450−500 bp	
			
	# CNEs	% Nucleotides	# CNEs	% Nucleotides	# CNEs	% Nucleotides
Gene	Overlapping	Overlapping	Overlapping	Overlapping	Overlapping	Overlapping

ZEB2	14/14	83.17	19/19	91.56	5/5	92.45
TSHZ3	6/6	88.50	12/12	89.36	2/2	90.68
EBF3	6/6	78.46	8/8	83.91	3/3	82.21
BCL11A	10/10	90.73	4/4	83.49	5/5	88.04
ZFHX4	6/6	93.58	5/5	93.10	6/6	87.98

#### 3.1.1 Genomic distribution of CNEs along the chromosome

**Fig. 1.**
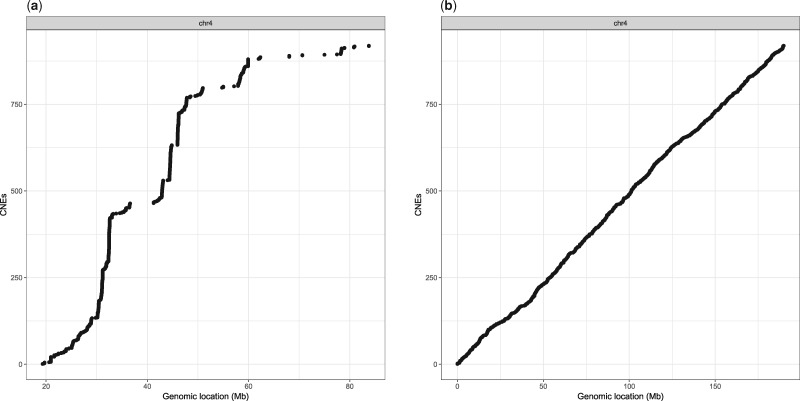
Genomic distribution of CNEs along human (hg38) chr4. (**a**) Elements found by CNEFinder. (**b**) CNE-like elements. For more information, see the text

We also wanted to find out whether the elements returned by CNEFinder are true CNEs in the biological sense. CNEs are known to form clusters in genomes (Sandelin *et al.*, 2004a), and the distances between consecutive elements follow power-law-like distributions (Polychronopoulos *et al.*, 2014b, 2016). We plotted the genomic distribution of human CNEs as identified by CNEFinder between Chromosome 4 (chr4) of the Human (hg38) and Chicken (galGal4), with t=90% and ℓ=50 bp. As a control, we also plotted the same number of human CNE-like elements; that is elements that have one by one, the same length as every CNE in the real set but are distributed randomly on chr4. The function plotCNEDistribution from the CNEr R/Bioconductor package (Tan, 2017) was used to produce the plots in Figure 1. Evidently from Figure 1, in the case of elements identified by CNEFinder, many elements are clustered around the same genomic position, while in the case of the control elements, this is clearly the contrary. The latter demonstrates that the elements identified by CNEFinder are indeed CNEs as they display an important biological property.

#### 3.1.2 Efficiency of CNEFinder

We also conducted the following typical runs to demonstrate the time and memory efficiency of CNEFinder. First, we recorded the time taken to compute CNEs with minimum and maximum length (bp), 200–250, 250–300, 300–350, 350–400, 400–450, and 450–500, with t=90%, between the 143–148 Mbp region of Chromosome 2 of the Human (hg19) genome and the 34–39 Mbp region of Chromosome 7 of the Chicken (galGal3) genome using eight CPU cores. These were, respectively, 4.4s, 4.4s, 4.5s, 4.8s, 4.3s, and 5.2s. The maximum memory used for these runs was 1.6 GB of RAM. Second, we recorded the time taken to compute CNEs with minimum and maximum length 200–500 bp with t=90% using the whole Chromosome 2 of the human (hg19) genome and the whole Chromosome 7 of the chicken (galGal3) genome using eight CPU cores. This was 32 m 30 s. The maximum memory used for this run was 5.6 GB of RAM.

#### 3.1.3 Comparison with local-alignment tools

In the last experiment, we exhibit the need for a tool specifically tailored for CNE identification. To this end, we compared CNEFinder to YASS, a state-of-the-art local alignment search tool (Noé and Kucherov, 2005). YASS works by identifying seeds between a pair of DNA sequences and then extends these matches to *local alignments* between the sequence pair. We ran YASS using regions 76.57–79.01 Mbp of Chromosome 8 of the Human (hg19) genome and 123.57–124.82 Mbp of Chromosome 2 of the Chicken (galGal3) genome. These are the exact regions used to compute the CNEs for gene ZFHX4 found in Table 1. A dissimilarity threshold of 5% was used to make the experiments as similar as possible. For the elements identified by YASS that did overlap with those in the UCNEbase, the average percentage of overlapping nucleotides was only 31.01%. This can be explained by the optimality criterion of local alignments that does not allow for constraints on the lengths of the alignments. Note that a comparison with other local-alignment tools is beyond the scope of this paper. The rationale of this experiment was to demonstrate the inapplicability of local alignment techniques for CNE identification.

## 4 Conclusion

Due to the lack of published tools for identifying CNEs and the need to systematically investigate their roles in genomes, we have presented CNEFinder, a tool specifically tailored for CNE identification given two DNA sequences. CNEFinder does not require or compute the whole-genome alignment or whole-genome indexes of the two sequences. It thus finds CNEs from the two sequences directly with user-defined criteria. Experimental results provided here show the accuracy of CNEFinder, compared to existing well-established static databases, as well as its efficiency and ability to reveal biological CNE trends on a chromosome level.
